# Perceived Threat and Internet Use Predict Intentions to Get Bowel Cancer Screening (Colonoscopy): Longitudinal Questionnaire Study

**DOI:** 10.2196/jmir.9144

**Published:** 2018-02-07

**Authors:** Daniela Becker, Johannes Grapendorf, Hannah Greving, Kai Sassenberg

**Affiliations:** ^1^ Social Processes Lab Leibniz-Institut für Wissensmedien Tübingen Germany; ^2^ Knowledge Construction Lab Leibniz-Institut für Wissensmedien Tübingen Germany; ^3^ University of Tübingen Tübingen Germany

**Keywords:** emotion, Internet, colonoscopy, cancer screening

## Abstract

**Background:**

Many people use the Internet for health-related information search, which is known to help regulate their emotional state. However, not much is known yet about how Web-based information search together with negative emotional states (ie, threat of cancer diagnosis) relate to preventive medical treatment decisions (ie, colonoscopy intentions).

**Objective:**

The aim of this study was to investigate how frequency of health-related Internet use together with perceived threat of a possible (bowel) cancer diagnosis influences intentions to get a colonoscopy. Previous research has shown that people who experience threat preferentially process positive information in an attempt to downregulate the aversive emotional state. The Internet can facilitate this regulatory strategy through allowing self-directed, unrestricted, and thus biased information search. In the context of threat regarding a possible bowel cancer diagnosis, feelings of threat can still be effectively reduced through cancer screening (ie, colonoscopy). We, therefore, predict that in that particular context, feelings of threat should be related to stronger colonoscopy intentions, and that this relationship should be enhanced for people who use the Internet often.

**Methods:**

A longitudinal questionnaire study was conducted among healthy participants who were approaching or just entering the bowel cancer risk group (aged 45-55 years). Perceived threat of a possible (bowel) cancer diagnosis, frequency of health-related Internet use, and intentions to have a colonoscopy were assessed at 2 time points (6-month time lag between the 2 measurement points T1 and T2). Multiple regression analyses were conducted to test whether threat and Internet use at T1 together predicted colonoscopy intentions at T2.

**Results:**

In line with our predictions, we found that the threat of a possible (bowel) cancer diagnosis interacted with the frequency of Internet use (both T1) to predict colonoscopy intentions (T2; *B*=.23, standard error [SE]=0.09, *P*=.01). For people who used the Internet relatively often (+1 SD), the positive relationship between threat and colonoscopy intentions was significantly stronger (*B*=.56, SE=0.15, *P*<.001) compared with participants who used the Internet less often (−1 SD; *B*=.17, SE=0.09, *P*=.07). This relationship was unique to Web-based (vs other types of) information search and independent of risk factors (eg, body mass index [BMI] and smoking).

**Conclusions:**

The results of this study suggest that health-related Internet use can facilitate emotion-regulatory processes. People who feel threatened by a possible (bowel) cancer diagnosis reported stronger colonoscopy intentions, especially when they used the Internet often. We propose that this is because people who experience threat are more likely to search for and process information that allows them to downregulate their aversive emotional state. In the present case of (bowel) cancer prevention, the most effective way to reduce threat is to get screened.

## Introduction

### Motivation to Undergo a Colonoscopy

Colonoscopy is a highly diagnostic tool for early bowel cancer detection and secondary prevention [1,2]. Health professionals, therefore, strongly encourage people at risk (eg, aged >50 years) to get screened. Despite the clear benefits, many people are still reluctant to have a colonoscopy [3]. Therefore, it is very important to understand the factors that motivate or discourage people to undergo a colonoscopy.

Until now, research on the predictors of colonoscopy attendance has focused predominantly on sociodemographic factors [4,5]. Recently, it has been suggested, however, that affective or emotional factors also play a key role in patients’ decision making [6]. This is—among other things—because negative emotional states have a significant and biasing influence on information processing and behavior [7]. Given that “cancer” is associated with negative emotions such as threat [8], colonoscopy attendance should also be affected by the threat associated with the illness.

Such emotion-based processing biases assert a particularly strong influence when information processing is self-directed and not guided by feedback or other means, as in case of Internet searches for health information [9]. Given that a large number of people are nowadays using the Internet to gather health-related information (72% of US Internet users [10]), it is important to understand the impact of Internet use on medical decision making. However, the effect of Internet use on medical decisions in highly affect-laden domains such as cancer prevention (ie, colonoscopy attendance) has so far mostly been neglected. In this study, we, therefore, investigated the joint influence of cancer-related threat and frequency of Internet use on people’s intentions to have a colonoscopy.

### Emotion Regulation on the Internet

When people use the Internet for health information search, they do not only pick up practical information (eg, what to do when having a cold) but they also regulate their emotional states (eg, finding comfort and relief by browsing through health-related forums [11]). Such emotion regulation in the context of Internet use is particularly likely in the health context because negative emotions such as threat are very common there. Patients can feel threatened by their diagnosis and its consequences for their everyday life, and healthy people can also feel threatened by the possibility of being diagnosed in the future. Regarding cancer, people report strong negative associations and emotions independent of whether they are diagnosed [8]. Those negative emotions can have a profound influence on how people perceive and process health-related information. Specifically, according to the principle of counter-regulation [12], people preferably search and process positive information when they feel threatened because positive information can help downregulate their negative emotional state. Particularly relevant to the current context, previous research on health-related Internet searches showed in the support of the counter-regulation principle that under threat, people search, process, and remember more positive information [13,14]. This positivity bias could be conceptually replicated in a longitudinal study among chronically ill patients. Here, the severity of patients’ disease (as a proxy of threat) predicted more positive perceptions of their own health 7 months later. Importantly, this relationship got stronger the more frequently patients used the Internet for health-related information searches, but not for those who used other sources [9]. This suggests that frequent Internet use augmented patients’ positivity bias, presumably because the Internet allowed them to (repeatedly) select positive information.

The positivity bias observed in the above studies suggests that people who feel threatened engage in emotion-focused coping to relieve their negative affective state [15]. Emotion-focused coping is a common and adaptive form of emotion regulation in situations in which control is low and people cannot do much about the emotion-eliciting situation—for instance, in case of a chronic illness as in the study summarized above [9]. However, emotion-focused coping is not always adaptive. A positivity bias could also make people underestimate the severity of their medical condition, which in turn can lead to suboptimal medical choices. Especially in situations in which people can still take preventive measures, such as colonoscopy, to reduce the risk of further developing a certain disease, emotion-focused coping and the implied positivity bias (“I am sure I am not affected anyway”) could have detrimental consequences. In fact, in those preventive health situations, people should be more likely to engage in problem-focused coping because this coping strategy is the more adaptive form of emotion regulation in situations in which people feel that there is a chance of restoring the lack of control (eg, being in a risk group but not yet affected). Accordingly, research has shown that in more controllable situations, people tend to directly address and change the emotion-eliciting situation rather than merely trying to feel better about it [15-17].

When feeling threatened by the possibility of receiving a cancer diagnosis, individuals should, therefore, look for ways to effectively reduce their threat. Given that a colonoscopy can help to regain certainty, and in the worst case receive a treatment at an early stage, it represents an effective means to reduce threat. Accordingly, people fearing bowel cancer should be particularly likely to get screened. Using the Internet to search for information regarding bowel cancer should further strengthen this link because numerous sites clearly communicate the high diagnosticity of colonoscopy and encourage making use of its benefits. Moreover, because the Internet allows self-directed and repeated information searches, people who use the Internet relatively often for health-related purposes and who strive to reduce their threat are more likely to engage with this positive and encouraging information. In that case, people’s regulatory needs (to reduce threat) are optimally supported by the self-directed and autonomous nature of Internet search. In contrast, exposure to offline information about colonoscopy (eg, through general practitioner, friends and brochures) should not have the same supportive effect on people’s intentions as offline information does not allow for a similar degree of self-directedness, autonomy, and the resulting selectivity [9].

However, to date, it is not yet clear how people’s feelings of threat regarding a possible cancer diagnosis are related to problem-focused coping strategies, such as increased intentions to have a colonoscopy, and whether Internet use has the predicted augmenting effect. Finding out more about the factors which make or keep people from going to cancer screenings is of pivotal importance, because cancer screening is a very effective tool for early cancer detection and, in the case of colonoscopy, there is not much risk attached to the screening itself [1]. Nevertheless, many people find a colonoscopy invasive and unpleasant, and those negative affective attitudes have previously been shown to be negatively related to people’s intentions to undergo a colonoscopy, as well as to their actual screening behavior [18,19]. Importantly, previous studies have either focused on global affective associations (eg, relaxed, tense, and happy [19]) or negative emotions regarding the procedure itself (eg, fear of pain or discomfort [18]). As argued above, we predict, however, that threat associated with a cancer diagnosis should be an independent and positive predictor of intentions to undergo a colonoscopy because it may motivate specific and more adaptive emotion-regulation strategies (ie, problem-focused coping).

This study investigates the role of perceived threat (of being diagnosed with bowel cancer) and people’s frequency of health-related Internet use on their intentions to have a colonoscopy. To test this, we recruit healthy participants aged between 45 and 55 years (no current cancer diagnosis or chronic disease), because in this age group, people should start to consider getting a colonoscopy in the near future. We predict that higher threat levels together with frequent health-related Internet use should enhance people’s intentions to get a colonoscopy. This positive relationship between threat and intentions should be less pronounced for people who use the Internet less often for health-related purposes.

To provide some background information for the main findings, we also plan to conduct the following additional analyses. First, we test whether our model holds when controlling for variables that have been shown to influence colonoscopy intentions in previous studies (ie, demographic factors [4,5] and threat of screening [18]). Second, we conduct exploratory analyses addressing further questions: does Internet use—as argued above—help to cope with the perceived threat of a diagnosis? To this end, we test whether Internet use predicts a reduction of perceived threat of a diagnosis over time. Does—in line with our argument—only Internet use moderate the impact of perceived threat of a diagnosis on colonoscopy intentions, or do the same effects occur for offline information search? And finally, how does the perceived threat of a diagnosis and its interaction with Internet use relate to risk factors of bowel cancer [20]?

## Methods

### Overview and Study Design

This study employed a longitudinal design with 2 measurement points (T1 and T2), which were approximately 6 months apart. We recruited a convenience sample of participants between the age of 45 and 55 years because people in this age group will face the decision whether or not to get a colonoscopy in the near future. Recruitment and measurement were done via a Web-based questionnaire, which ensured that our sample was at least somewhat familiar with using the Internet in a personal health context. Our main predictor variables (threat of cancer diagnosis and frequency of Internet use) as well as our outcome variable (colonoscopy intentions) were assessed with multiple-item self-report measures.

### Participants

Participants were recruited via different Web-based platforms for a study related to psychology, health, and cancer prevention. The study was conducted in German language and described as a survey on cancer screening, and targeted a group of participants aged between 45 and 55 years. Participation was voluntary and the only exclusion criteria were a (current or past) cancer diagnosis or a diagnosis of a chronic disease (eg, diabetes). Completing the survey at both measurement points was rewarded with a voucher worth €10. This study was approved by the ethical committee of the Faculty of Medicine at the University of Tübingen, Germany.

### Main Questionnaire

Upon following an link, participants were guided to an introduction page, which included information about the study (duration, reward, purpose of the study, ethical approval, and anonymity of data handling). Once participants had read the information, they could give informed consent by ticking a box. To ensure complete anonymity of the data handling process, participants provided their email address, which was stored separately and could neither be accessed by the researchers nor later be matched to their personal data. The system administration then sent an email with the link to the survey, which was administered via the Web-based survey program Qualtrics. In addition, 6 months later, another email with the link to the second survey was sent automatically and independent of whether participants had completed the first measurement. All email addresses were deleted after data collection had ended. To receive the voucher, participants had to re-enter their email address at the end of the questionnaire in the second measurement point, which was again stored separately from the questionnaire data.

The questionnaire was pretested by healthy participants. To increase user friendliness, a process bar was displayed throughout the survey, and the number of items presented per page was adjusted in such a way that scrolling was mostly unnecessary. After answering (and if necessary correcting) all questions presented on one page, participants clicked on a “next” button. Participants were not able to return to already completed pages.

At the beginning of both measurements, participants generated a unique code, which would later allow matching their data without violating their anonymity. After receiving an overview of the questionnaire, participants reported their age, gender, and highest level of education. The main part of the survey contained the measurement of the key variables (see below) but also several additional measures. (This study was conducted in collaboration with another laboratory, and the additional measures were included to test their hypotheses. To increase transparency, the list with additional measures can be found in Multimedia Appendix 1.) As T1 contained more items than T2, T1 took on average 31 min and 55 s (SD 11.58, n=150) and T2 took 20 min and 5 s (SD 8.57, n=150) to complete.

### Measures

#### Threat of Diagnosis

Our main predictor was participants’ perceived level of threat regarding a possible cancer diagnosis. In addition, we assessed participants’ perceived level of threat regarding the screening itself (ie, colonoscopy) [18], to be able to distinguish between the 2 different types of threat. Both types of threat were measured with respect to the following: (1) cancer in general and (2) bowel cancer in particular. There were in total 15 items measuring threat (diagnosis and screening) of general cancer and 15 items measuring threat (diagnosis and screening) of bowel cancer. All threat items were modeled after Peacock and Wong’s [21] stress appraisal measure, and contained estimates of stress, threat, anxiety, outcome negativity, and helplessness. Threat regarding (bowel) cancer diagnosis was assessed with items such as “I am afraid of being diagnosed with (bowel) cancer.” Threat regarding a (bowel) cancer screening was assessed with items such as “Undergoing cancer screening (a colonoscopy) would be a threatening situation for me.” Responses were given on 5-point Likert scales (1=*not at all applicable* and 5=*very applicable*). Factor analyses confirmed the 2 subtypes (threat diagnosis vs screening) for both general and bowel cancer (general cancer: eigenvalue_T1_ 2.73 vs 6.17, eigenvalue_T2_ 2.40 vs 5.93; bowel cancer: eigenvalue_T1_ 3.50 vs 5.17, eigenvalue_T2_ 2.82 vs 5.13). One nonfitting item was dropped. High intercorrelations (*r*_150_>.8) between general and bowel cancer items suggested that they should be treated as one construct (additional analyses showed that the main model presented below holds when only looking at threat regarding bowel cancer). This resulted in the following two final variables: our main predictor variable threat of cancer diagnosis (6+6 items; alpha_T1_=.87, alpha_T2_=.85) and the additional variable threat of cancer screening (8+8 items; alpha_T1_=.95, alpha_T2_=.95).

#### Internet Use

The second predictor of interest was health-related Internet use and was assessed with 2 self-report items (for a similar procedure see [9]). One item asked about the frequency of general health-related Internet use on a 7-point scale (“How often do you use the Internet for health-related purposes?”; 1=*several times a day*, 2=*once a day*, 3=*2-5 times a week*, 4=*once a week*, 5=*1-2 times a month*, 6=*2-6 times a year*, and 7=*rarely or never*), and about the frequency of Internet use regarding information on cancer prevention on a 5-point Likert scale (“Have you used the Internet to gather information on cancer prevention?”; 1=*never* and 5=*a lot*). We pooled those 2 items (reverse-coded and converted the 7-point scale into a 5-point scale) to arrive at a general Internet use variable (*r*_150,T1_=.63, *P*<.001; *r*_150,T2_=.41, *P*<.001).

#### Colonoscopy Intentions

Participants’ intentions to get a colonoscopy were measured by the following 2 questions (assessed on 5-point Likert scales): (1) whether they would participate when asked by the doctor (1=*I would not participate* and 5=*I would participate*), and (2) whether they would actively ask for it (1=*definitely* and 5=*under no circumstances*). Both items were averaged (after reverse-coding the second item) to form the main outcome variable (*r*_150,T1_=.22, *P*=.01; *r*_150,T2_=.44, *P*<.001).

#### Additional Variables

We assessed the following 7 risk factors for bowel cancer based on the clinical literature [20]: family member with a bowel cancer diagnosis (“Are there are instances of bowel cancer known in your family” *yes* vs *no)*, current diagnosis of bowel disease (“Are you currently diagnosed with a bowel disease?”; *yes* vs *no*), smoking (“Are you smoking?”; *yes* vs *no*), quality of diet (“How would you evaluate your diet?”; 1=*poor* and 5=*balanced*), quality of lifestyle (“How would you evaluate your lifestyle?” 1=*poor* and 5=*balanced*), physical activity (“How many minutes per week do you exercise?”; 1=*less than 30 min,* 2=*more than 30 but less than 60 min,* 3=*more than 60 but less than 90 min,* 4=*more than 90 but less than 120 min,* 5=*more than 120 but less than 150 min,* and 6=*more than 150 min*), and body mass index (BMI; weight [kg]/height [m]×height [m]).

We also estimated the degree to which participants use other sources than the Internet to get information on health-related issues (“Which other sources of information do you use, and how often?”). Responses were given on 5-point Likert scales (1=*rarely* and 5=*very regularly*) for the following options: general practitioner, therapist, family or friends or acquaintances, books, television or radio, newspaper or magazine, and public events or lectures. Those were combined to a single average score reflecting the use of alternative information sources (alpha_T1_=.72, alpha_T2_=.61).

#### Data Analysis

**Figure 1:**
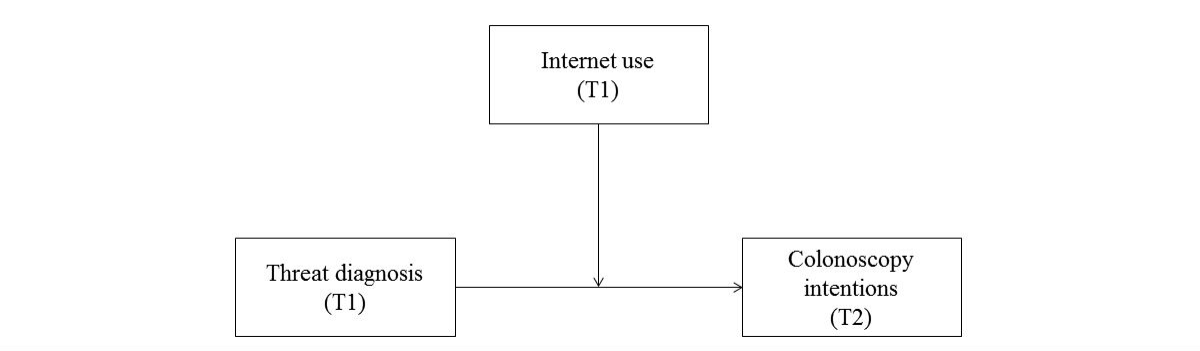
The main model tested in our analyses.

To test the main hypothesis that people’s colonoscopy intentions are predicted by an interaction of their perceived threat of a diagnosis and their Internet use (Figure 1), a multiple regression analysis was conducted. Colonoscopy intention (T2) was regressed on threat of diagnosis, Internet use (both T1), and their interaction, as well as colonoscopy intentions at T1 (autocorrelation). All predictor variables were mean-centered; the outcome variable was left in its original metric. Before carrying out the respective regression analyses, we computed the correlations among the predictor variables to check for multicollinearity.

In the second step, we tested separately whether the predicted interaction between perceived threat of a diagnosis and Internet use explains colonoscopy intentions above and beyond the known predictors: (1) sociodemographic variables [4,5] and (2) threat of screening [18]. Therefore, we conducted two additional multiple regressions in which these known predictors (both assessed at T1) and perceived threat of diagnosis (T1), Internet use (T1), and their interaction were entered as predictors (and the autocorrelation) of colonoscopy intentions (T2).

Moreover, we conducted exploratory analyses to test the additional research questions mentioned above. First, to test whether Internet use contributes to coping with the perceived threat of a diagnosis, we regressed perceived threat of the diagnosis at T2 on Internet use at T1, perceived threat of diagnosis at T1, and their interaction. Second, we also tested whether offline information search moderated the effect of perceived threat of diagnosis on colonoscopy intentions just as Web-based information search. Here, we conducted a multiple regression predicting colonoscopy intentions (T2) by offline information search (T1), perceived threat of a diagnosis (T1), and the interaction between the last two factors (and the autocorrelation). Finally, to test the role of actual risk factors, we computed their correlation with perceived threat of a diagnosis in bivariate correlations. Moreover, to find out whether perceived threat asserts an effect beyond the actual risk factors, we entered the risk factors as additional predictors into the regression conducted to test the main prediction. All tests were conducted with Statistical Package for the Social Sciences (SPSS) version 22 (IBM Corp).

## Results

### Sample Description and Dropout Analysis

**Figure 2:**
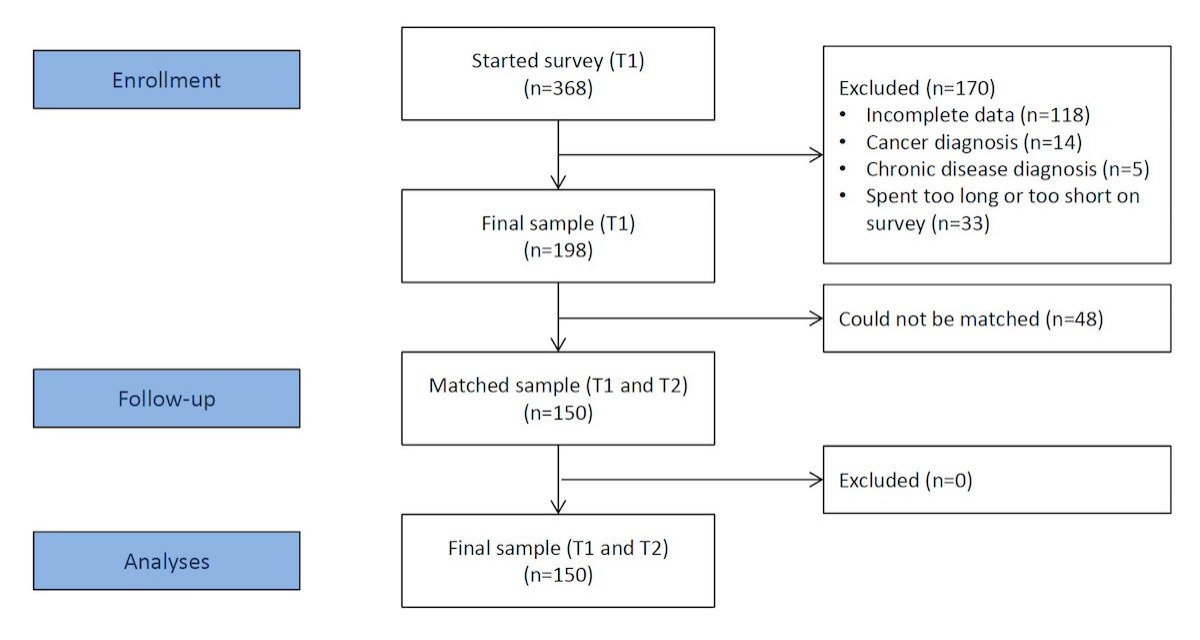
Generating the final sample.

Of the 368 participants who started the questionnaire at T1, 250 participants completed it (ie, answered all mandatory questions: 250/368, 67.9%). Following our a priori exclusion criteria, we excluded 14 participants who were diagnosed with cancer and 5 people who indicated to be diagnosed with a chronic disease (diabetes; n_new_=231). We additionally excluded 33 participants who spent less than 10 or more than 60 min on the survey because their responses were likely unreliable (mean time of completion of the final sample was 31 min and 55 s; n_new_=198). From those 198 datasets, 150 could be matched with a completed and reliable questionnaire at T2 (150/198, 75.8%): dropout rate of 24.2% (48/198) from T1 to T2 (see Figure 2).

Participants who dropped out did not differ from the final sample with respect to gender (χ^2^_1,n=198_=1.10, *P*=.29) but were slightly older (mean=49.73 years, SD 3.27) than the final sample (mean=48.44 years, SD 2.93; *t*_196_=2.58, *P*=.01, *d*=0.43).

Participants who were included in the final sample (vs dropout) reported slightly lower threat levels with regards to a possible cancer diagnosis (mean=3.36 years, SD 0.59 vs mean=3.67 years, SD 0.81; *t*_63.61_=2.46, *P*=.02, *d*=0.48) and used the Internet more often for health-related purposes (mean=3.40 years, SD 0.84 vs mean=3.02 years, SD 1.08; *t*_62.80_=−2.23, *P*=.03, *d*=0.42). Despite the differences between the subsamples, we remain confident that our final sample is suitable for analysis because the differences we found are rather small and such differences are mostly problematic in contexts of intervention testing (eg, randomized controlled trials), but much less so in longitudinal questionnaire studies such as this study.

The final sample consisted of 150 participants (60.7% female; mean=48.44 years, SD 2.93, min=45 years, max=55 years). Participants’ highest obtained level of education was relatively high, with the majority of people (102/150, 68.0%) holding a certificate of secondary education after 10 years of schooling. A total of 21 participants (14.0%) were holding at least a high school degree (ie, 12 years of schooling), and 27 participants (18.0%) held a university (polytechnic) degree (see Table 1 for an overview of sample characteristics).

Of all participants, the minority reported incidents of bowel cancer in their family (14/150, 9.3%). Moreover, only a few participants had already gotten a colonoscopy (T1: 12/150, 8.0%; T2: 14/150, 9.3%). Excluding participants who had already undergone colonoscopy from the sample did not significantly alter the results reported below.

### Test of Predictions

In the main regression model, we predicted people’s colonoscopy intentions (at T2) with their perceived threat of a cancer diagnosis (at T1) and their Internet use (at T1; see Figure 1). Participants’ colonoscopy intentions at T1 were also included in the model (autocorrelation). Multicollinearity checks revealed that our main predictor threat of diagnosis was uncorrelated with Internet use (for intercorrelations, see Table 2).

**Table 1:** Sample characteristics of the final sample (N=150) at T1.

Characteristic		Value
Age in years, mean (SD)		48.44 (2.93)
**Gender, n (%)**		
	Female	91 (60.7)
	Male	59 (39.3)
**Education (finished), n (%)**		
	Secondary education	102 (68)
	High school	21 (14.0)
	University degree	27 (18.0)
Family cancer history, n (%)		14 (9.3)
Bowel disease diagnosis, n (%)		5 (3.3)
Smoking, n (%)		8 (5.3)
Body mass index, mean (SD)		22.90 (3.01)
Quality of diet, mean (SD)		2.25 (0.79)
Quality of lifestyle, mean (SD)		2.31 (1.00)
Physical activity, mean (SD)		3.54 (1.20)

**Table 2:** Pearson correlations between measures used in the regression analysis (N=150) and descriptive.

Measure	Threat diagnosis (T1)	Internet use (T1)	Colonoscopy intentions (T1)	Mean (SD)
Threat diagnosis (T1)	-	-	-	3.36 (0.59)
Internet use (T1)	−.13	-	-	3.40 (0.84)
Colonoscopy intentions (T1)	.13	−.11	-	3.37 (0.78)
Colonoscopy intentions (T2)	.26^a^	−.15^b^	.55^c^	3.44 (0.78)

^a^*P*<.01.

^b^*P*<.10.

^c^*P*<.001.

The regression model testing the main hypothesis was significant (adj. *R*^2^=.36, standard error [SE]=0.63, *F*_4,145_=21.48, *P*<.001; see Table 3). Threat of diagnosis was related to stronger colonoscopy intentions (*B*=.37, SE=.10, *P*<.001), whereas Internet use in and of itself was unrelated (*B*=−.10, SE=0.06, *P*=.11).

**Figure 3:**
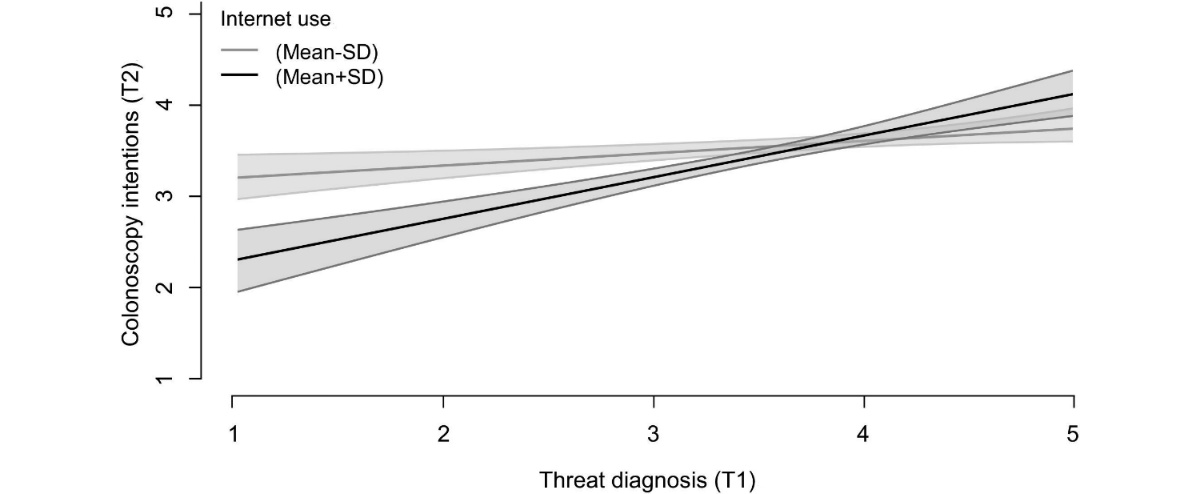
The relationship between threat of diagnosis (T1) and colonoscopy intentions (T2) as a function of participants’ frequency of Internet use (T1; at +1 SD and −1 SD). Shaded areas represent the ±1 standard error margin.

Most importantly, the predicted interaction reached significance (*B*=.23, SE=.09, *P*=.01). In line with our prediction, simple slope analyses showed that for participants with relatively high Internet use (+1 SD), threat of cancer positively predicted intentions to participate in colonoscopy (*B*=.56, SE=.15, *P*<.001), whereas this relation was marginal for participants with low Internet use (−1 SD; *B*=.17, SE=.09, *P*=.07; see Figure 3).

In the second regression analysis, we again tested the main model but also controlled for main effects of major sociodemographic factors such as age, gender, and education level. None of the factors were significant predictors in the model, and including them did not influence the original predicted interaction between threat and Internet use (Table 3).

In the third regression analysis, we extended the main model with the main effect of threat of screening (T1) because previous research had identified the threat of screening as a predictor of screening intentions [18]. Replicating previous findings, threat of screening predicted reduced intentions to have a colonoscopy (*B*=−.21, SE=0.08, *P*=.01; Table 3). The original predicted interaction between threat of diagnosis and Internet use remained marginally significant (*B*=.17, SE=0.09, *P*=.07). Simple slope analyses showed a similar pattern as in the main model for participants with high Internet use (+1 SD), threat of diagnosis was positively related to intentions (*B*=.48, SE=0.15, *P*=.002). This was also, but much less so, the case for participants who use the Internet less often (−1 SD, *B*=.19, SE=0.09, *P*=.04).

### Exploratory Analyses

#### Internet Use and Coping

In the introduction, we argued that Internet use can augment coping processes that aim at reducing negative emotions such as threat. In an exploratory analysis, we tested whether threat of diagnosis at T1 together with Internet use at T1 predicted threat of diagnosis at T2. The respective regression model was significant (adj. *R*^2^=.69, SE=.31, *F*_3,146_=111.70, *P*<.001). Besides a significant autocorrelation between the two threat measures (*B*=.75, SE=0.05, *P*<.001), we also obtained a main effect of Internet use (*B*=−.14, SE=0.03, *P*<.001), which suggested that the more people used the Internet at T1 for health-related purposes, the lower their threat levels at T2. Hence, using the Internet appears to be an efficient means of coping with threat related to bowel cancer.

#### Source of Information

In the next analysis, we tested whether the moderating role of Internet use is unique to Internet use, or whether searching alternative offline sources (eg, magazines, television, and friends) for health-related information has a similar effect. To test this, we replaced the predictor Internet use with the measure of participants’ use of alternative sources. Besides the autocorrelation, the only significant predictor was threat of diagnosis (*B*=.29, SE=0.10, *P*=.004). There was neither a main effect of alternative information use (*P*=.40) nor an interaction with threat (*P*=.22). This suggests that the effects are assumed specific to Internet use.

#### Risk Factors

In the final set of analyses, we tested whether it is the “right” (ie, at risk) people that feel threatened. From the 7 risk factors, 3 were significantly correlated to participants’ level of threat at T1: smoking (smokers felt more threatened by diagnosis; *r*_150_=.25, *P*=.002), having a family member who has had bowel cancer (participants with a cancer diagnosis in the family felt more threatened; *r*_150_=.20, *P*=.01), and BMI (the higher the BMI, the stronger the threat; *r*_150_=.20, *P*=.02).

Adding all 7 risk factors as additional predictors to the main regression model shows that none of the risk factors were themselves related to colonoscopy intentions (all *P* s>.05). Moreover, controlling for those factors did not alter the interaction between threat of diagnosis and Internet use (*B*=.23, SE=0.10, *P*=.02). These results indicate that participants’ threat of cancer partly relies on risk factors, but people’s emotions and not these risk factors contribute to the intention to participate in cancer screening.

**Table 3:** Parameter estimates from different regression analyses predicting colonoscopy intentions (T2).

Model and predictors			*B*	Standard error	*t*	*F*	Degrees of freedom	Adjusted *R*^2^
**Main model**						21.48^a^	4,145	.36
	Threat diagnosis (T1)		.37	.10	3.61^a^			
	Internet use (T1)		−.10	.06	−1.63			
	Threat×Internet use		.23	.09	2.57^b^			
	Colonoscopy intentions (T1)		.52	.07	7.75^a^			
**Second model**						12.60^a^	7,142	.35
	Threat diagnosis (T1)		.34	.11	3.12^c^			
	Internet use (T1)		−.10	.07	−1.39			
	Threat×Internet use		.23	.09	2.55^b^			
	Colonoscopy intentions (T1)		.51	.07	7.44^a^			
	**Demographic variables (T1)**							
		Age	.01	.02	0.43			
		Gender	−.14	.11	−1.25			
		Education	.04	.04	0.86			
**Third model**						19.19^a^	5,144	.38
	Threat diagnosis (T1)		.34	.10	3.37^c^			
	Internet use (T1)		.01	.08	0.17			
	Threat×Internet use		.17	.09	1.84^d^			
	Colonoscopy intentions (T1)		.42	.08	5.42^b^			
	Threat screening (T1)		−.21	.08	−2.58^b^			

^a^*P*<.001.

^b^*P*<.05.

^c^*P*<.01.

^d^*P*=.07.

## Discussion

### Principal Findings

The goal of this longitudinal study was to investigate the role of perceived threat of cancer and people’s frequency of health-related Internet use on their intentions to have a colonoscopy. In support of our predictions, we found that higher levels of threat together with frequent health-related Internet use increased people’s intentions to have a colonoscopy 6 months later. Importantly, this relationship was independent of demographic factors (ie, gender, age, and level of education) and factors associated with the risk of developing bowel cancer (eg, BMI and smoking). It was also unique to Web-based (other types of) information search. Our findings are, therefore, in line with (1) the coping literature suggesting that in situations in which control over negative emotions such as threat and uncertainty is still possible (eg, cancer prevention through colonoscopy), people are more likely to engage in adaptive problem-focused coping [15]; (2) previous research showing that frequent health-related Internet use can augment such coping processes [9]; and (3) the recent appeal that emotional factors are key predictors of medical decision making and should therefore be investigated more thoroughly [6].

We propose that our findings can best be explained within the coping literature, according to which negative emotions such as threat and uncertainty motivate people to engage in behavior that reduces this aversive emotional state. In situations in which control can be restored, such as in the context of cancer prevention (eg, colonoscopy), people should be most likely to engage in problem-focused coping and search for ways to effectively reduce their threat. Given the high diagnosticity of colonoscopy and the possibility of early detection and intervention, it represents a suitable means to regain certainty and reduce threat. For people who use the Internet often for health-related information search, the benefits of colonoscopy should become even more salient. That is because most information about colonoscopy on the Web univocally emphasizes its benefits and encourages people in the risk group (eg, from 50 years onwards) to get screened, and because the Internet allows people to align their search behavior with their current emotional needs (ie, reduce threat). Our main findings, as well as our exploratory finding that enhanced Internet use reduced feelings of threat 6 months later, suggest that with its combination of encouraging content and self-directed search behavior, the Internet provides an optimal context for (problem-focused) coping to take place.

Although we did—in line with our expectations—not find parallel effects for offline and Web-based information search, similar effects can certainly also occur offline. The counter-regulation resulting in selective information processing [12-14] underlying the reported effect is not limited to a specific type of information source. It becomes more likely, however, when multiple sources provide the basis for selective information processing. Thus, when an individual discusses with many people whether or not to get screened, we may obtain similar results, given the majority of information is encouraging. As the Internet offers a large amount of easily accessible, predominantly encouraging information, it would be much more difficult to collect the same amount and type of information offline. Therefore, we deem the reported effects to be more likely to result from Web-based rather than from offline information search.

The finding that the perceived threat of diagnosis is related to (self-reported) risk factors suggests that the perceived threat is not irrational. However, given that the interaction between threat and Internet use predicts colonoscopy intentions beyond the risk factors, the current findings emphasize the notion that emotions play an important role in medical decision making [6]. However, research investigating emotional predictors of colonoscopy intentions and attendance is still scarce. Moreover, the few existing studies reported predominantly negative effects [18,19]. For example, threat regarding the colonoscopy screening itself had been associated with reduced intentions to get screened. In this study, we did not only replicate this previous finding but we also extended it by showing that threat regarding a possible cancer diagnosis has the opposite positive effect on colonoscopy intentions—especially when people used the Internet often for health-related questions. This study, therefore, contributes to a more nuanced understanding of the different ways in which negative emotions can impact medical decision making.

### Limitations and Future Directions

One limitation of this study is that we measured intentions rather than real colonoscopy attendance. That was partly because of the choice of our sample (aged 45 to 55 years), which was just entering the phase in which preventive cancer screening gains importance. Accordingly, only a small number of participants had actually had colonoscopy at T2 (14/150, 9.3%). Nevertheless, studying intentions still offers valuable insights because intentions are direct antecedents of behavior. More specifically, intentions capture the commitment and motivational inclination toward a specific behavior, and several studies suggest that they are indeed reliable predictors of the implied behavior [22,23]. Future research in an older sample is, however, needed to test whether emotional factors together with Internet use have a similar effect on actual screening behavior.

Another point of discussion concerns the question of what information people are actually searching for and processing when browsing the Internet for health-related purposes. In this study, we only assessed the frequency of their search behavior, but not the actual content. Measuring real search behavior would have been difficult to realize given this study’s longitudinal questionnaire design. To reliably measure Web-based information search, additional studies with higher experimental control would be required. Such studies already exist in the context of threat, Internet search, and emotion-focused coping (eg, people search, process, and remember more positive information in a state of threat [13,14]), but similar studies extending those findings to the domain of problem-focused coping are still lacking.

Nevertheless, our exploratory finding that Internet use at T1 reduced feelings of threat at T2 sheds some light on the intraindividual dynamics underlying our main finding. This finding suggests that people processed information that helped regulate their negative emotions. This interpretation seems likely considering that most of the available information on the Web stresses the benefits and high diagnosticty of the screening method. An important question for future research is, however, whether a similar effect could be obtained for medical decisions, which are not as beneficially portrayed on the Internet (eg, prostate cancer screening). In such cases, threatened people who use the Internet more frequently for health-related information search may get even more uncertain about a specific (preventive) medical intervention, which in turn could lower their intentions. It is, therefore, important to emphasize that for now, the current findings should be interpreted in the specific context of bowel cancer screening (ie, colonoscopy).

Finally, our Web-based sampling procedure may have biased the pattern of our results because we oversampled Internet users. Although many people are using the Internet for health-related purposes [10], especially older people (who are at risk) tend to use the Internet less. Although this suggests that the sample is biased regarding these demographic factors, it does not question the generalizability of the current findings because they focused specifically on Internet users. Another aspect of our sampling procedure should also be considered. As the study was explicitly announced as study on cancer prevention, the sample may mainly consist of people who were already interested in this topic. Although it seems difficult to predict how this affected the results, further research should aim at replicating the current findings with a more representative sample.

### Implications

In this study, we demonstrated that negative emotions such as feelings of threat can have a significant influence on screening intentions. Our study, therefore, extends previous work by showing that negative emotions do not only keep people from getting screened [18,19] but they can also motivate people to get screened, provided they activate problem-focused coping strategies. This is important information for general practitioners or anyone concerned with increasing colonoscopy attendance rates. Although it may be beneficial to reduce people’s threat regarding the screening procedure itself [18], our findings suggest that reducing or ignoring people’s threat regarding a possible cancer diagnosis may lower its potential to instigate problem-focused coping strategies. Instead, it may be more effective to take people’s feelings of threat seriously, and respond to them by stressing that a colonoscopy can help reduce uncertainty because of its high diagnosticity and possibility for early cancer treatment. This implication seems warranted, independent of whether information is communicated via the Web or offline.

Our findings also highlight the important role of Internet use in the health context. It seems that frequent health-related Internet use augmented people’s coping efforts and screening intentions. This is an important finding because Internet use in the health context has often been associated with negative aspects, such as low quality of information and inaccurate self-diagnoses [24]. This study, however, shows that Internet use in the health context can also be beneficial because it strengthened cancer screening intentions among those who feel most threatened by a possible diagnosis. As mentioned above, this finding should be interpreted in the specific context of colonoscopy, as we do not know yet how information on the Web about less positively portrayed medical interventions may interact with people’s emotional states and their coping strategies.

Interestingly, for those who reported low levels of threat, frequent Internet use was related to weaker intentions. This could either be because those people are less likely to search for information concerning bowel cancer screening in the first place, or because they simply do not engage with it in the same emotionally oriented way people with stronger feelings of threat would do. This would imply that health information on the Web may be most (or only) effective when it matches people’s emotional and regulatory needs. In other words, emotional states such as threat can act as a catalyst amplifying the message of Web-based health information. Although this had positive consequences in the present context of cancer prevention, the same mechanism may also be responsible for less positive phenomena such as cyberchondria [25]. Here, Internet use amplifies people’s anxiety regarding a possible diagnosis so that they become increasingly convinced that they are sick. It is important to note, however, that cyberchondria is an example of maladaptive coping, whereas in this study, we were mainly interested in adaptive coping. That the Internet can contribute to both is an important conclusion because cyberchondria and other negative effects often dominate the public debate.

### Conclusions

This longitudinal study showed that people’s emotional states interact with their health-related Internet use in predicting screening intentions. More specifically, we found that colonoscopy intentions were highest among people who reported strong feelings of threat regarding a (bowel) cancer diagnosis and who use the Internet often for health-related information search. We propose that this is because information on the Web about colonoscopy is predominantly positive, highlighting the diagnosticity of the screening method, and because people who experience threat are likely to preferentially and repeatedly process that specific type of information [13], as it can help reduce their aversive state of threat. Internet search in the health domain may therefore represent one way through which emotion regulation can be facilitated.

## Appendix

Multimedia Appendix 1 List of additional scales administered as part of this study.

## Abbreviations

BMI: body mass index
SPSS: Statistical Package for the Social Sciences
SE: standard error
T1: first measurement point
T2: second measurement point
