# Supplementary material for: Perceived Threat and Internet Use Predict Intentions to Get Bowel Cancer Screening (Colonoscopy): Longitudinal Questionnaire Study
Source: J Med Internet Res. 2018 Feb 7;20(2):e46. doi: 10.2196/jmir.9144 (PMC5822037; doi:10.2196/jmir.9144)
Supplement: Multimedia Appendix 1 [file jmir_v20i2e46_app1.pdf]

Multimedia Appendix 1

List of additional scales administered as part of the present study. German translations were used. Some scales were shortened. Reliabilities based on N = 150.

Scale name	# items	Alpha T1	Alpha T2
**Positive and Negative Affect Schedule (PANAS) [1]**			
**Negative Affect**	6	.918	.851
**Positive Affect**	6	.625	.586
**Self-control [2]**	5	.604	.560
**Regulatory focus [3]**			
**Prevention**	5	.320	-
**Promotion**	5	.416	-
**Health-related Self-esteem [4]**	7	.563	.555
**Hypochondria [5]**	7	.892	.837
**Health-related Self-efficacy [6]**	6	.328	.357
**Attitude to Alternative Medicine [7]**	5	.590	.599
**Attitude to Conventional Medicine [7]**	6	.773	.735
**Motivation relating to Bowel Cancer Screening [8]**	11	.817	.741
**Epistemic beliefsrelating to Bowel Cancer Screening [9]**	17	.629	.635
**John Hopkins Symptom Checklist [10]**	25	.972	.957
**Individual Health Concept [11]**			
Evidence based medicine	4	.654	.746
Patient-centered medicine	4	.629	.745
**Patients' needs for Bowel Cancer Screening [12]**			
**Need for clarity**	3	.731	-
Need for well-being	3	.638	-
Need for reliability	2	.504	-
**Patients' needs relating to medical consultation**	13	.923	-

[1] Watson, D., Clark, L. A., & Tellegen, A. (1988). Development and validation of brief measures of positive and negative affect: the PANAS scales. *Journal of Personality and Social Psychology*, 54(6), 1063.

[2] Rheinberg, F. & Wendland, M. (2003). Itemübersicht zum Fragebogen SSI-K-32. Potsdam: Universität Potsdam, Institut für Psychologie.

[3] Sassenberg, K., Ellemers, N., & Scheepers, D. (2012). The attraction of social power: The influence of construing power as opportunity versus responsibility. *Journal of Experimental Social Psychology*, *48*(2), 550-555.

[4] Heatherton, T. F., & Polivy, J. (1991). Development and validation of a scale for measuring state self-esteem. *Journal of Personality and Social Psychology*, *60*(6), 895.

[5] Pilowsky, I. (1993). Dimensions of illness behavour as measured by the Illness Behaviour Questionnaire: A replication study. *Journal of Psychosomatic Research*, *37*, 53–62.

[6] Sallis, J. F., Pinski, R. B., Grossman, R. M., Patterson, T. L., & Nader, P. R. (1988). The development of self-efficacy scales for health-related diet and exercise behaviors. *Health Education Research, 3,* 283-292.

[7] Furnham, A., & Forey, J. (1994). The attitudes, behaviors and beliefs of patients of conventional vs. complementary (alternative) medicine. *Journal of Clinical Psychology*, *50*(3), 458-469.
[8] Pelletier, L. G., Tuson, K. M., & Haddad, N. K. (1997). Client motivation for therapy scale: A measure of intrinsic motivation, extrinsic motivation, and amotivation for therapy. *Journal of Personality Assessment*, *68*(2), 414-435.
[9] Stahl, E., & Bromme, R. (2007). The CAEB: An instrument for measuring connotative aspects of epistemological beliefs. *Learning and Instruction*, *17*(6), 773-785.
[10] Derogatis, L. R., Lipman, R. S., Rickels, K., Uhlenhuth, E. H., & Covi, L. (1974). The Hopkins Symptom Checklist (HSCL): A self-report symptom inventory. *Systems Research and Behavioral Science*, *19*(1), 1-15.
[11] Bientzle, M., Cress, U., & Kimmerle, J. (2015). The role of tentative decisions and health concepts in assessing information about mammography screening. *Psychology, Health & Medicine, 20*, 670-679.
[12] Bientzle, M., Fissler, T., Cress, U., & Kimmerle, J. (2017). The impact of physicians' communication styles on evaluation of physicians and information processing: A randomized study with simulated video consultations on contraception with an intrauterine device. *Health Expectations, 20*, 845-851.
